# The mechanisms how heparin affects the tumor cell induced VEGF and chemokine release from platelets to attenuate the early metastatic niche formation

**DOI:** 10.1371/journal.pone.0191303

**Published:** 2018-01-18

**Authors:** Jan Moritz Ponert, Svenja Schwarz, Reza Haschemi, Jens Müller, Bernd Pötzsch, Gerd Bendas, Martin Schlesinger

**Affiliations:** 1 Department of Pharmacy, Rheinische Friedrich-Wilhelms-University Bonn, Bonn, Germany; 2 Institute for Experimental Hematology and Transfusion Medicine, University of Bonn Medical Centre, Bonn, Germany; Institut d'Investigacions Biomediques de Barcelona, SPAIN

## Abstract

Metastasis is responsible for the majority of cancer associated fatalities. Tumor cells leaving the primary tumor and entering the blood flow immediately interact with platelets. Activated platelets contribute in different ways to cancer cell survival and proliferation, e.g. in formation of the early metastatic niche by release of different growth factors and chemokines. Here we show that a direct interaction between platelets and MV3 melanoma or MCF7 breast cancer cells induces platelet activation and a VEGF release in citrated plasma that cannot be further elevated by the coagulation cascade and generated thrombin. In contrast, the release of platelet-derived chemokines CXCL5 and CXCL7 depends on both, a thrombin-mediated platelet activation and a direct interaction between tumor cells and platelets. Preincubation of platelets with therapeutic concentrations of unfractionated heparin reduces the tumor cell initiated VEGF release from platelets. In contrast, tumor cell induced CXCL5 and CXCL7 release from platelets was not impacted by heparin pretreatment in citrated plasma. In defibrinated, recalcified plasma, on the contrary, heparin is able to reduce CXCL5 and CXCL7 release from platelets by thrombin inhibition. Our data indicate that different chemokines and growth factors in diverse platelet granules are released in tightly regulated processes by various trigger mechanisms. We show for the first time that heparin is able to reduce the mediator release induced by different tumor cells both in a contact and coagulation dependent manner.

## Introduction

The tumor microenvironment has a crucial impact on tumor cell survival, proliferation and metastasis. Next to components of the extracellular matrix, various cells have been identified in the tumor tissue that increase tumorigenicity by inhibiting the antitumor immune responses [1–3]. Furthermore they contribute to angiogenesis by secreting angiogenic factors [4], or expedite tumor cell extravasation by inducing an epithelial to mesenchymal transition in the tumor cells [5–7]. Especially for the process of hematogenous metastasis, the leading cause for cancer related death and major number of fatalities, a vital support of tumors by other cells is indispensable. After leaving the primary tumor and entering the blood circulation, tumor cells immediately interact with blood components creating a hospitable microenvironment [8]. Monocytes, macrophages and neutrophils are mostly described to be recruited to the early metastatic foci [9–14], supporting metastatic dissemination in different ways, e.g. by increasing tumor cell extravasation, preventing tumor cell lysis by NK cells, or transmitting survival signals to the tumor cells [15,16]. Chemokines like CCL2, CCL5 or G-CSF, among many others, which are secreted by the tumor or endothelial cells [9,11–13], are responsible for leukocyte attraction.

Besides leukocytes, platelets are the major components interacting at first (within 2–5 minutes) with the tumor cells entering the blood [17,18]. Platelets immediately surround the tumor cells, thereby protecting them from shear forces of the blood and NK cell based immune responses [19–22]. Additionally, platelets have the capability to induce an EMT program in tumor cells [23] by converting the epithelial to a more mesenchymal phenotype. Cells which have passed through an EMT program have acquired traits of cancer stem cells, which is accompanied by elevated malignancy [24,25]. Platelets are also involved in the recruitment of granulocytes to the tumor cell-platelet-agglomeration by secretion of chemokines CXCL5 and CXCL7, which activate the granulocyte expressed receptor CXCR2. Recruited granulocytes contribute to tumor cell extravasation from the blood [26]. Finally, platelets, associated to and activated by tumor cells secret vascular endothelial growth factor (VEGF) which creates a proangiogenic environment [27].

Heparin has been considered as a promising pharmacological approach to interfere with the metastatic spread of tumors in addition to its guideline-based application in terms of anticoagulant prophylaxis or treatment of cancer patients. Preclinical data confirm that heparin can interfere with metastatic spread as a multi-target drug, e.g. affecting tumor cell adhesion or migration [28,29]. A recent study reported that preincubation of platelets with heparin induced a mitigated platelet-derived VEGF release whereas the angiostatic endostatin secretion was augmented [27]. Obviously, heparin seems also to be able to modulate tumor cell induced mediator release from platelets to an angiostatic ratio. Nevertheless, underlying mechanisms and the relevance for other mediators than VEGF remain to be elucidated.

In the present study, we investigate the impact of unfractionated heparin on platelet-derived VEGF, as well as CXCL5 and CXCL7 release. Surprisingly, platelets were activated by tumor cells due to a juxtacrine/contact-dependent signaling which could efficiently be blocked by heparin. Associated with reduced contact between tumor cells and platelets, the VEGF secretion was reduced by heparin preincubation. In contrast, the release of CXCL5 and CXCL7 was not affected by a heparin preincubation of the platelets but could be further enhanced by activation of the coagulation cascade. We reveal that different platelet-derived mediators are released by different mechanisms, which can only partially be blocked by heparin. Our data indicate that platelet mediator release due to tumor cell contact is an intricate process, which goes far beyond the current paradigm of platelet-derived mediator release.

## Methods

### Cell lines

Human MV3 melanoma cell line [30] and human myelomonocytic U937 [31] cells were cultivated in RPMI 1640 medium (PAN Biotech, Aidenbach, Germany) containing 10% (v/v) fetal calf serum (FCS) (Sigma Aldrich, Steinheim, Germany), 100 U/mL penicillin and 100 μg/mL streptomycin (PAN Biotech). EA.hy926 endothelial cells were cultured in Dulbecco’s modified Eagle’s Medium (DMEM low glucose) (Sigma Aldrich) with 10% FCS, 100 U/mL penicillin and 100 μg/mL streptomycin [32]. MCF-7 breast cancer cells were maintained in DMEM (high glucose) medium and supplemented with 10% FCS, 1% L-glutamine, 100 U/mL penicillin and 100 μg/mL streptomycin [33]. All cells were incubated at 37°C in a humidified atmosphere containing 5% (v/v) CO_2_. For subcultivation, MV3, MCF7 and EA.hy926 cells were detached at a confluency of about 90% with EDTA-solution (0.2 g/L EDTA x tetra sodium, Sigma Aldrich) for 5 min at 37°C. Test for absence of mycoplasms were performed routinely every month.

### Blood collection, platelet preparation and activation, plasma preparation

Human blood was collected in accordance to the declaration of Helsinki from 5 healthy volunteers who did not ingest aspirin or nonsteroidal anti-inflammatory drugs or other drugs influencing platelet function for at least 10 days prior to blood draw. Ethical approval was obtained from the ethics committee of Medical Center Bonn (070/05). Blood donors/participants provided their written consent to participate in the study.

Blood from the antecubital vein was drawn into citrate tubes (10.5 mM final concentration, Sarstedt, Nümbrecht, Germany).

Prior to centrifugation, tubes were stored for a maximum time of 1 hour at room temperature and platelet-rich-plasma (PRP) was obtained by centrifugation at 200 × g for 20 min. Platelets were counted by using a Neubauer improved counting chamber. Usually 2 × 10^8^ platelets/mL were used for the following assays. Prior to activation, in some experiments, platelets were preincubated with different anticoagulants for 30 min. The anticoagulant exposure was performed in PRP using unfractionated heparin (UFH, obtained from ratiopharm GmbH, Ulm, Germany) to achieve a concentration of 1 I.U./mL or fondaparinux adjusted to a concentration of 775 ng/mL in PRP (Aspen Pharma, Dublin, Irland). Concentrations of unfractionated heparin as well as fondaparinux correspond to therapeutic concentrations in anticoagulated patients. Both anticoagulants were diluted in water. Anti-human P-selectin mAb (R&D Systems, Wiesbaden Nordenstadt, Germany) was applied at a concentration of 1 μg/mL in PRP. Platelets were activated with 35 μM ADP (Sigma-Aldrich), 10 μM Thrombin Receptor Activating Peptide-6 (TRAP-6) (Tocris Bioscience, Bristol, UK), 5 × 10^5^ MV3 human melanoma cells/100 μL PRP or 5 × 10^5^ MCF7 breast cancer cells/100 μL PRP in the presence or absence of anticoagulants for 12 min at 37°C. To obtain releasates, samples were centrifuged for 20 min at 1000 × g and supernatants collected. In order to generate defibrinated plasma, platelet-poor-plasma (PPP) was incubated for 20 min at 37°C with batroxobin (Siemens Healthcare, Erlangen, Germany). Afterwards, the clotted fibrin was separated from the PPP by centrifugation. To ensure total defibrination of the PPP, a second batroxobin incubation was performed for 20 min at 37°C and newly formed fibrin was again removed by centrifugation. The defibrinated PPP was stored at -20°C until used. In order to recalcify plasma, CaCl_2_ to a final physiological concentration of 2.5 mM was added.

### Platelet tumor cell adhesion

To quantify tumor cell platelet interactions, MV3 or MCF7 cells were grown confluently in 96-well plates. Platelets were labeled with Calcein-AM at a final concentration of 2 μM for 15 min at 37°C and washed with warm (37°C) PBS buffer, afterwards. Tumor cells were washed twice with warm (37°C) PBS buffer and 5 × 10^7^ Calcein-AM-labeled platelets (Sigma-Aldrich) in 100 μL PPP were added together with anticoagulants or anti-P-selectin mAb and platelet activators in some cases. After shaking for 15 min, unbound platelets were removed by washing twice with warm PBS. Platelets bound to tumor cells were lysed with 100 μL Triton X-100 (10% in PBS) and transferred to black 96-well plates. Fluorescence was quantified at an excitation wavelength of 485 nm and an emission wavelength of 520 nm using a plate reader (BMG POLARstar, BMG Labtech, Ortenberg, Germany).

### Fluorescence microscopy

Tumor-platelet interaction was visualized by fluorescence microscopy using an Axiovert 200 microscope (Carl Zeiss, Oberkochen, Germany). Calcein-labeled platelets (5 × 10^7^ in 100 μL PPP) were added to confluent layers of tumor cells (MV3 or MCF7 cells) in 96-well plates in combination with anticoagulants and activators, respectively. After washing twice with warm PBS, representative areas of each well were monitored by fluorescence microscopy.

### Tube formation assay

Platelets were isolated from PRP by centrifugation for 20 min at 1000 × g. The platelet pellet was resuspended in platelet buffer (10 mM N-2-hydroxyethylpiperazine-N9-2-ethanesulfonic acid, 140 mM NaCl, 3 mM KCl, 0.5 mM MgCl_2_ 2.5 mM NaHCO_3_, 10 mM glucose) and platelets were washed with platelet wash buffer (140 mM NaCl, 5 mM KCl, 12 mM trisodium citrate, 10 mM glucose, 12.5 mM sucrose, pH = 6.0) to remove remaining citrate. The platelet pellet was resuspended in platelet buffer and the concentration was adjusted to 2 × 10^8^ platelets/mL. Samples were incubated for 30 min with anticoagulants and activated with ADP, TRAP-6 or tumor cells, respectively. Platelet supernatants were collected by centrifugation for 20 min at 1000 × g. In parallel, 96-well plates were coated with geltrex (Thermo Fisher Scientific, Langenselbold, Germany) for 30 min at 37°C and 3.5 × 10^4^ EA.hy926 human endothelial cells and platelet releasate, VEGF or no supplements, respectively, were added per well. Tube formation was evaluated by branch point counting after 24 h of incubation. Representative areas were imaged with an Axiovert 200 microscope (Carl Zeiss). Results of independent assays were averaged and for the testing of statistical significance, the Students t-test was applied.

### Granulocyte transmigration assay

To characterize the impact of heparin on platelets´ chemokine release, a boyden chamber transmigration approach with 3 μm pores was applied. Neutrophils were isolated from EDTA blood using a MACSxpress isolation kit according to manufacturer´s instructions (Miltenyi Biotec, Bergisch Gladbach, Germany). Neutrophils (2 × 10^5^ in 50 μL RPMI medium containing no FCS) were pipetted on the upper part of the membrane of the chemotaxis transmigration chamber (ChemoTx System, Neuro Probe, Gaithersburg, USA). In the bottom of the chamber, platelet releasates (from platelets treated with heparin and activators, respectively) were added and the transmigration plate was incubated for 2 h at 37°C. Afterwards, transmigration plate was centrifuged in a plate centrifuge for 5 min at 350 × g. The transmigrated neutrophils in the bottom part of the chamber were transferred to a white 96-well plate and subsequently quantified by the CellTiter-Glo Luminescent Cell Viability Assay (Promega, Mannheim, Germany) using a microplate reader (BMG POLARstar, BMG Labtech).

### Thrombin generation assay

The ability of MV3 and MCF7 tumor cells to induce thrombin formation and subsequent platelet activation in plasma was assessed using a microplate-based, fluorogenic thrombin generation assay (calibrated automated thrombography [CAT]) [34]. Briefly, 20 μL PBS containing 4 x 10^5^ MV3 or MCF7 tumor cells or 20 μL of tissue factor reagent (5 pM or 1 pM final concentration; Stago, Düsseldorf, Germany) were added to 80 μL PPP, substituted with corn trypsin inhibitor (FXIIa inhibitor, 30 μg/mL, Santa Cruz Biotechnology, Heidelberg, Germany) to attenuate the activation of the intrinsic coagulation pathway. After addition of 20 μL of starting solution (containing CaCl_2_ for recalcification) and the fluorogenic peptide substrate Z-Gly-Gly-Arg-AMC (Bachem, Weil am Rhein, Germany), the kinetics of thrombin-mediated substrate hydrolysis were monitored at 37°C using a Fluoroskan Ascent plate reader (Thermo Fisher Scientific). In some experiments, PRP or PPP lacking Factor VII were used. Furthermore, some experiments were performed in the absence of CaCl_2_. Each experiment was performed in triplicate.

### Flow cytometry

To determine platelet activation, 100 μL of PRP, containing 1 × 10^7^ platelets were incubated with ADP, TRAP-6, or MV3 melanoma cells (1 × 10^6^), respectively, for 10 min. FITC-labeled anti-P-selectin mAb (R&D Systems) or FITC-fibrinogen (Molecular Probes, Leiden, The Netherlands) were added to the platelets and incubated for 30 min on ice. Expression of P-selectin or binding of FITC-fibrinogen to the integrin α_IIb_β_3_ on platelets was determined by flow cytometry (FACScalibur, BD Biosciences, Heidelberg, Germany). Expression of Tissue Factor on tumor cells was measured by flow cytometry after incubation of tumor cells with FITC-labeled anti-TF mAbs (R&D Systems).

### VEGF, CXCL5 and CXCL7 ELISAs

The VEGF, CXCL5 and CXCL7 concentrations in platelet releasates were quantified at least in triplicates using human enzyme-linked immunosorbent assays (ELISAs) according to the manufacturer’s instructions. CXCL5 and CXCL7 ELISAs were purchased from R&D Systems while the VEGF ELISAs were from PeproTech (Rocky Hill, NY, USA). Samples for the VEGF ELISAs were applied at 4-fold dilution, the samples for the CXCL5 ELISA at 8-fold dilution and samples for the CXCL7 ELISA at 4000-fold dilution. Each dilution was made with the assay specific diluent.

### Statistical analysis

Data represent means ± standard deviations of at least three independent experiments if not indicated otherwise. Student’s t-test was applied for statistical analysis. P<0.05 was considered statistically significant and marked with a star. Two stars indicated a p-value below 0.01 and three stars were used for p-values below 0.001.

## Results

### Platelet-derived VEGF release due to direct tumor cell contact

**Fig 1:**
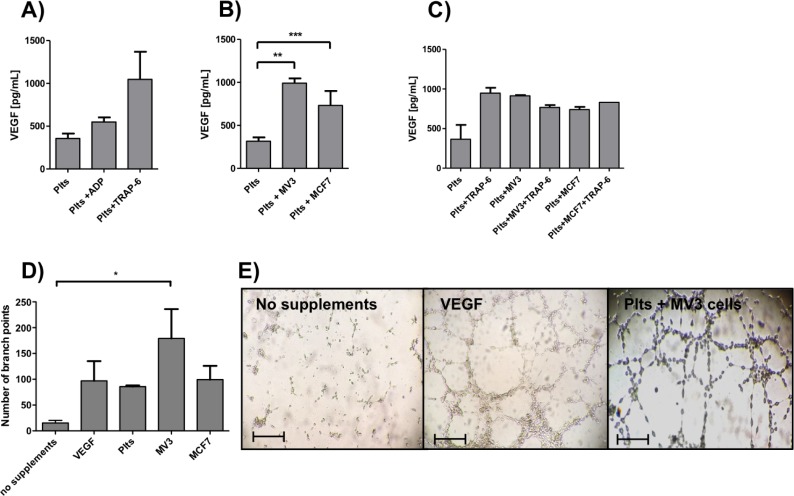
Tumor cell induced VEGF release. (A) Platelets in citrated plasma were incubated for 10 min with ADP and TRAP-6 and VEGF in plasma supernatant was determined by ELISA of at least 10 samples per treatment. (B) Platelets in citrated plasma were incubated for 12 min with MV3 and MCF7 cells, respectively, and VEGF in plasma supernatant was determined by ELISA of at least 10 samples per treatment. (C) Platelets were incubated for 12 min with TRAP-6, MV3 cells or a combination of both, respectively, and VEGF concentration in supernatant was quantified by ELISA. (D) EA.hy926 endothelial cells were grown on Matrigel with no supplements, VEGF, supernatant of unactivated platelets, or supernatant of platelets in citrated plasma treated before with MV3 or MCF7 cells, respectively. Number of branch points was determined after 24 h for each experiment. (E) Representative pictures of EA.hy926 cells incubated with VEGF, platelet releasate from platelets incubated with MV3 cells (for 12 min) previously, or no supplements, respectively. For VEGF and platelet releasate treated EA.hy926 cells, tube formation is detectable. Bars correspond to 100 μm.

Platelets contribute to the formation of a proper metastatic niche in different ways, e.g. by secretion of growth factors, which finally initiate the formation of new blood vessels in growing metastatic nodules. Tumor cells are capable to activate platelets and to induce a growth factor release by different modes. At first, the well-known platelet activators ADP and TRAP-6 were added to PRP and VEGF release was quantified by ELISA. Both, ADP as well as TRAP-6 elevated the VEGF concentration in the platelet releasate compared to untreated platelets from 350 pg/mL to 550 pg/mL or 1050 pg/mL VEGF, respectively (Fig 1A). To investigate the ability of MV3 melanoma and MCF7 breast cancer cells to induce a VEGF release from platelets by direct contact, we incubated platelets with tumor cells in citrated plasma and measured the platelet-derived VEGF release. MV3 and MCF7 cells triggered a pronounced VEGF secretion from platelets (Fig 1B), which could not be exceeded by additional administration of TRAP-6 to MV3 and MCF-7 cells, respectively (Fig 1C). Hence, the direct contact between platelets and tumor cells seems to be independent of thrombin or the coagulation cascade, which is not active in citrated PRP. To examine the functional relevance of the platelet-derived VEGF release due to tumor cell contact, tube formation assays were conducted. Supernatant from platelets previously treated with MV3 or MCF7 cells were added to endothelial cells and an increased number of branch points were detectable after 12 h of incubation (Fig 1D and 1E). In contrast, U937 monocytes were unable to induce a VEGF release from platelets after coincubation (S1A Fig). Thus, the direct interaction with tumor cells in the absence of plasmatic coagulation processes (and thrombin) seems to exert a strong effect on platelet granules containing VEGF.

### Platelet activation by tumor cells

**Fig 2:**
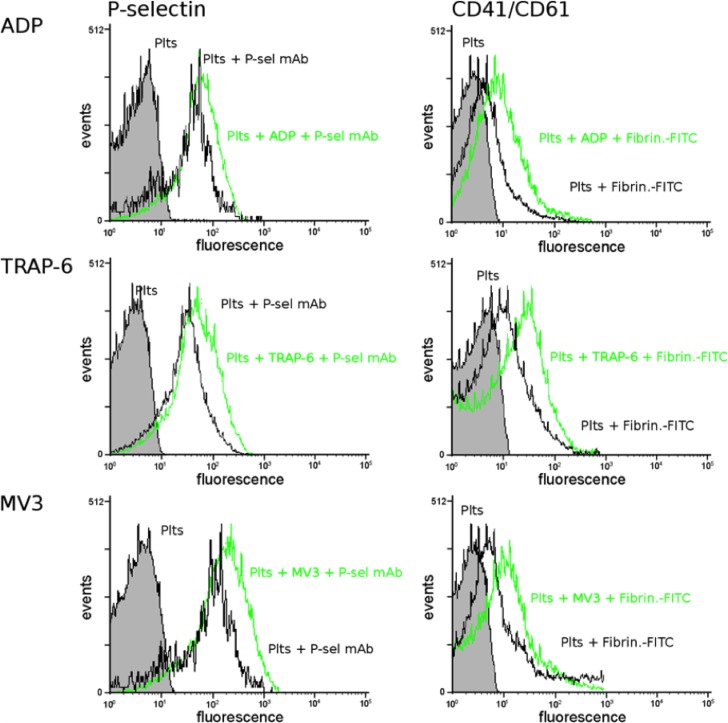
Tumor cell induced platelet activation. Platelets in citrated plasma were incubated with ADP, TRAP-6, or MV3 melanoma cells, respectively, and P-selectin or integrin α_IIb_β_3_ (CD41/CD61) were labeled either with anti-P-selectin mAbs or FITC-fibrinogen as ligand for activated integrin α_IIb_β_3_. P-selectin presentation and integrin α_IIb_β_3_ activation were determined by flow cytometry and compared to unstimulated platelets.

Because platelet secretion precedes platelet aggregation, we sought to determine expression of the adhesion receptor P-selectin and the affinity of the integrin α_IIb_β_3_ (CD41/CD61) to fibrinogen after tumor cell contact on the platelets´ surface. For this reason, platelets were incubated with ADP or TRAP-6 to stimulate the activation pathways driven by the P_2_Y_12_ receptor or thrombin-receptor PAR-1 and analyzed by flow cytometry. MV3 cells or aggregates of platelets and MV3 cells were distinguished by their sizes from single platelets. ADP, TRAP-6 as well as MV3 melanoma cells induced an increased P-selectin presentation on the platelets (Fig 2 left part) compared to untreated platelets. As a second marker for platelet activation, we investigated the binding affinity of platelet integrin α_IIb_β_3_ (CD41/CD61) to FITC labeled fibrinogen. Platelets exposed to ADP, TRAP-6, or MV3 cells, respectively, revealed an augmented binding capacity towards FITC-fibrinogen, as again analyzed by flow cytometry (Fig 2 right part). Our data show that MV3 melanoma cells have a comparable potency to induce platelet activation like ADP or TRAP-6, which activate the platelets via the P_2_Y_12_ receptor or the PAR-1 pathway, respectively.

### Interaction of platelets with tumor cells

**Fig 3:**
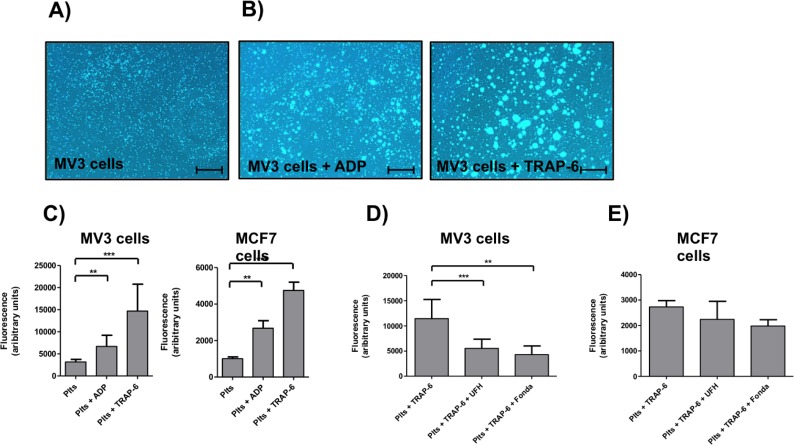
Interaction of tumor cells with platelets. (A) Representative pictures of Calcein-AM labeled platelets interacting with confluent layers of MV3 cells (black background). Scale bar 25 μm. (B) Representative pictures of ADP or TRAP-6 activated and Calcein-AM labeled platelets adhering to confluent layers of MV3 cells (black background). Scale bar 25 μm. (C) Quantification of platelet (Calcein-AM labeled) tumor cell (MV3 and MCF7) interaction with a plate reader. In some cases platelets were activated either with ADP or TRAP-6. (D) Impact of UFH or fondaparinux, respectively, on platelet (Calcein-AM labeled) MV3 cell interaction. (E) Impact of UFH or fondaparinux, respectively, on platelet (Calcein-AM labeled) MCF7 cell interaction.

A direct contact formation with tumor cells appears as dominant activation factor for platelets. To elucidate the involved mechanisms, we investigated the interaction of platelets with tumor cells by microscopic means. Platelets revealed a strong tendency to adhere to confluent layers of MV3 melanoma cells (Fig 3A) without further stimulus. However, this interaction could be further expedited by administration of platelet activators like ADP or TRAP-6 (Fig 3B and 3C). To elucidate the mechanisms of the strong platelet / tumor cell interaction, we blocked P-selectin on platelets by application of unfractionated heparin and fondaparinux, respectively. Unfractionated heparin binds to P- and L-selectin with high affinity and blocks the interactions with their counter receptors. However, heparin and fondaparinux impeded the binding between TRAP-6 activated platelets and MV3 cells whereas the interaction with MCF7 cells (Fig 3D and 3E) was hardly affected. Application of anti-human P-selectin mAb to platelets also reduced the binding between MV3 tumor cells and ADP or TRAP-6 activated platelets, respectively (S2A Fig).

### Impact of heparin on platelet-derived VEGF release

**Fig 4:**
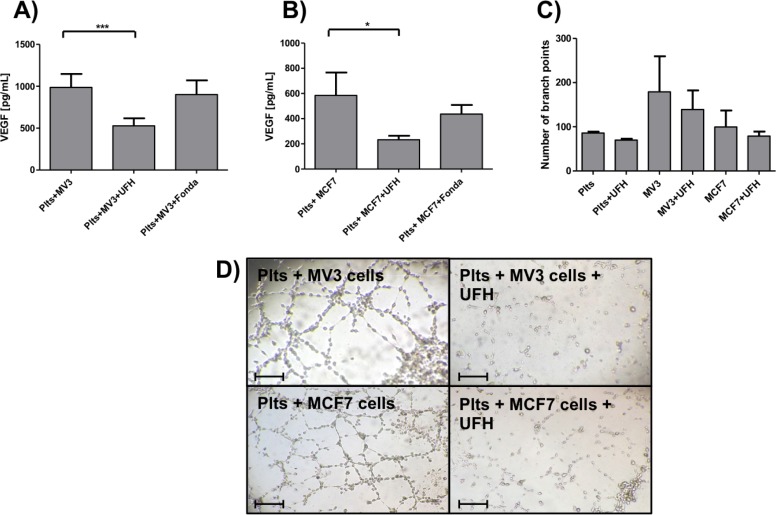
Impact of heparin on platelet-derived VEGF release. (A) Impact of UFH or fondaparinux, respectively, on MV3 cell induced VEGF release from platelets. (B) Impact of UFH or fondaparinux, respectively, on MCF7 cell induced VEGF release from platelets. (C) Impact of UFH on tube formation of EA.hy926 cells induced by MV3 and MCF7 mediated platelet activation. (D) Representative pictures of tube formation of EA.hy926 cells induced by releasates from platelets incubated with MV3 or MCF7 cells (for 12 min) previously. In some cases platelets were preincubated with UFH. Bars correspond to 100 μm.

Consequently, since heparin is able to interfere in the direct interplay between tumor cells and platelets, we next asked whether heparin is also able inhibit tumor cell induced VEGF release. Preincubation of platelets with UFH resulted in a bisection of VEGF concentration from 1000 pg/mL PRP to 500 pg/mL for MV3 melanoma cells (Fig 4A). Fondaparinux in contrast had hardly any effect on the VEGF release. MCF7 cell induced VEGF secretion was strongly affected by a preincubation with UFH. Fondaparinux reduced the VEGF release slightly on the contrary (Fig 4B). In order to evaluate, if P-selectin is involved in VEGF release from platelets due to tumor cell interaction, we applied anti P-selectin mAbs to platelets. For MV3 as well as MCF-7 cells, no impact on VEGF release could be detected due to P-selectin inhibition (S2B Fig).

Next, we tested whether the inhibitory capacity of heparin to reduce VEGF concentrations is functionally reflected in the formation of angiogenic tubes. For both cell lines, a platelet preincubation with UFH resulted in a reduced number of branch points, but these reductions in branch point formation were not significant compared to platelets solely incubated with tumor cells (Fig 4C and 4D).

### Impact of heparin on platelet-derived CXCL5 and CXCL7 release

Besides growth factors, platelets´ payload also consists of several different chemokines, which participate in the development of the early metastatic niche. For instance, the chemokines CXCL5 and CXCL7, derived from platelets, have been proven to recruit granulocytes to tumor cells circulating in the blood [26]. The granulocytes in turn confer survival and pro-tumorigenic signals to the tumor cells.

**Fig 5:**
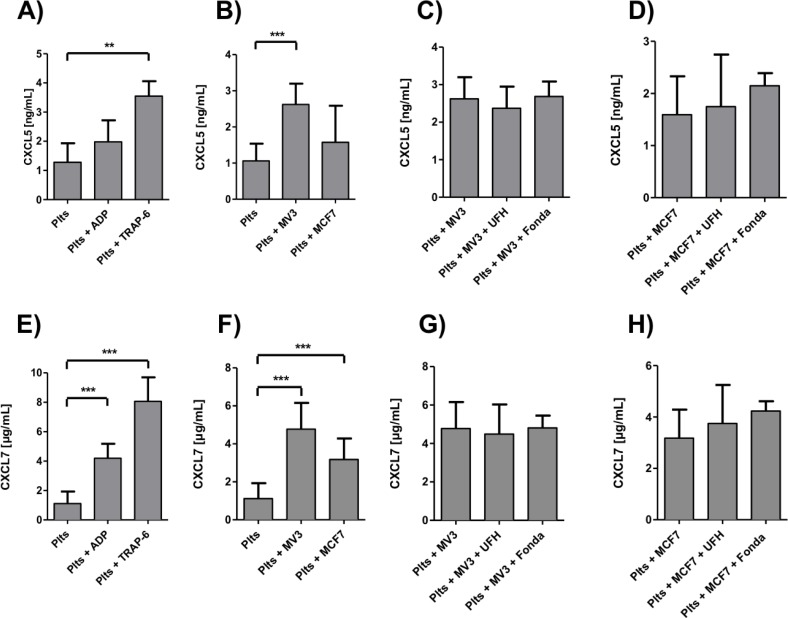
Tumor cell induced platelet-derived CXCL5 and CXCL7 release. (A) Platelets in citrated plasma were treated with ADP and TRAP-6 and CXCL5 release was quantified by ELISA. (B) Platelets were incubated with MV3 or MCF7 cells, respectively, and CXCL5 release was determined by ELISA. (C) Platelets, preincubated with UFH or fondaparinux, respectively, were incubated with MV3 cells. CXCL5 release was quantified by ELISA. (D) Platelets, preincubated with UFH or fondaparinux, respectively, were incubated with MCF7 cells. CXCL5 release was measured by ELISA. (E) Platelets were either treated with ADP or TRAP-6 and CXCL7 release was quantified by ELISA. (F) Platelets were incubated with MV3 or MCF7 cells, respectively, and CXCL7 release was determined by ELISA. (G) Platelets, preincubated with UFH or fondaparinux, respectively, were incubated with MV3 cells. CXCL7 release was determined by ELISA. (H) Platelets, preincubated with UFH or fondaparinux, respectively, were incubated with MCF7 cells. CXCL7 release was measured by ELISA.

Addition of ADP or TRAP-6 (Fig 5A), as well as MV3 and MCF7 cells (Fig 5B), respectively, induced an increased CXCL5 release from platelets. Comparable to VEGF, MV3 cells exhibited a stronger CXCL5 release than MCF7 cells. Preincubation of platelets with fondaparinux had no impact on CXCL5 concentration for MV3 (Fig 5C) as well as MCF7 cells (Fig 5D). Only UFH exerted marginal effects on the CXCL5 concentration. This stands in contrast to the VEGF release from platelets, which was clearly mitigated by UFH preincubation (Fig 4A and 4B).

Similar results were obtained for the secretion characteristics of platelet-derived CXCL7. The common platelet activators ADP and TRAP-6 augmented the CXCL7 secretion akin to concentrations achieved by MV3 or MCF7 incubation (Fig 5E and 5F). A 30 min preincubation of platelets either with UFH or fondaparinux had no impact on the CXCL7 release induced by MV3 or MCF7 cells (Fig 5G and 5H). Thus, the direct contact between tumor cells and platelets induces platelet activation and a chemokine release, which is not prone to a heparin preincubation. To enquire if granulocyte chemotaxis is influenced by heparin, we conducted neutrophil transmigration experiments, using a boyden chamber approach. Neutrophils migrating towards platelet releasates showed an augmented migration when platelets had been coincubated with MV3 cells. UFH preincubation again had only a very slight impact on neutrophil migration characteristics (S1B Fig).

### Tumor cell characterization concerning procoagulant properties

**Fig 6:**
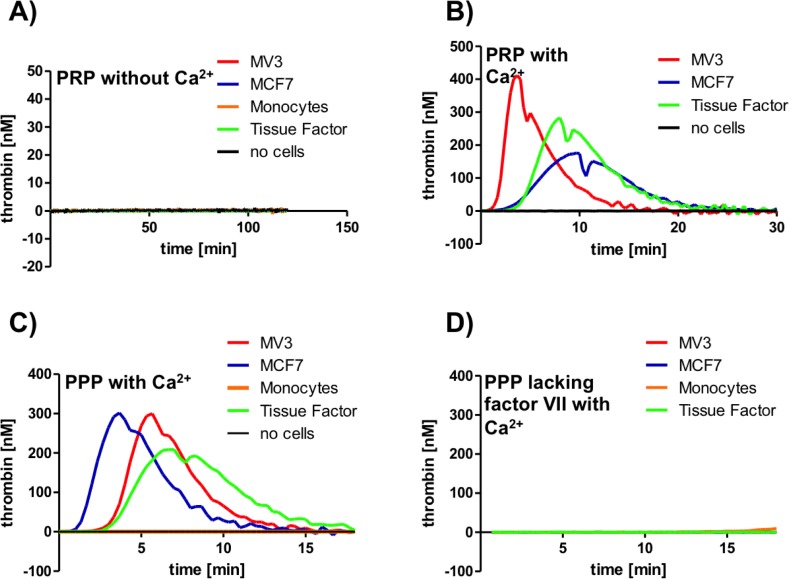
Tumor cell characterization concerning thrombin generation. (A) Thrombin generation of MV3, MCF7 cells and freshly isolated monocytes, respectively, was determined in platelet-rich plasma (PRP) without recalcification and compared to the effect of tissue factor (TF) (5 pM). No thrombin formation was detectable. (B) Thrombin generation of MV3, MCF7 cells was determined in recalcified PRP and compared to 5 pM TF. (C) Thrombin generation of MV3, MCF7 cells and monocytes, respectively, was quantified in recalcified PPP and compared to 5 pM TF. (D) Thrombin generation of MV3, MCF7 cells and monocytes, respectively, was quantified in recalcified Factor VII deficient PPP and compared to the effect of TF (5 pM).

Next to a direct contact formation, platelet activation triggered by the coagulation cascade and finally thrombin is described in the literature for several tumor cell lines [35]. To include this kind of potential platelet activation in our experimental settings we first characterized the mechanism by which MV3 melanoma and MCF7 breast cancer cells initiate platelet activation. Therefore we applied a plasma-based thrombin generation assay. Thrombin, the key enzyme of the plasmatic coagulation cascade, is able to activate platelets via cleavage of the N-terminus of platelets PAR-1 receptor. Citrated PRP was incubated with a fluorogenic substrate that is specifically cleaved by thrombin. However, MV3 melanoma, and MCF7 breast cancer cells did not induce a thrombin generation after 120 min in citrated PRP (Fig 6A). Hence, the tumor cell induced platelet activation in PRP, which we observed in the flow cytometry experiments (Fig 2) must accordingly be conveyed by a direct contact formation between platelets and tumor cells and not by factors of the coagulation cascade. To investigate in general whether the tumor cell possess procoagulant properties, we added calcium ions to the citrated PRP and could observe a pronounced thrombin generation for both tumor cell lines at a comparable level similar to tissue factor (TF) (5 pM final concentration), which served as a control (Fig 6B). Similar results were obtained in PPP in which calcium ions and MV3, or MCF7 cells were added. Here, both tumor cell lines were even more potent in activating thrombin generation than 5 pM TF (Fig 6C). As a further control, freshly isolated monocytes from human blood were unable to induce thrombin generation (Fig 6C).

In the next experiments we tried to elucidate which clotting factor, presented by the tumor cells, is responsible for the distinct thrombin generation. For this reason, we used PPP with factor VII deficiency and added calcium ions and either MV3 cells or MCF7 cells, or TF, respectively. Interestingly, no thrombin formation could be observed (Fig 6D). This indicates that the substantial thrombin formation of the tumor cell lines observed in recalcified PPP or PRP was initially induced by TF expressed by the tumor cells and not by tumor cell secreted thrombin. To address the question, if TF is localized on the tumor cell surface or secreted by the cells into the medium, we repeated the experiments and added tumor cell supernatant to recalcified PPP (S1C Fig). The supernatant failed to induce significant thrombin concentrations in PPP. Flow cytometry experiments finally revealed a TF expression on the cell membranes of both tumor cell lines (S1D Fig). In this respect, tumor cell expressed TF, which induces a pronounced thrombin formation, is localized on the tumor cell surfaces and not shed into the medium.

### Impact of heparin on coagulation dependent platelet activation

**Fig 7:**
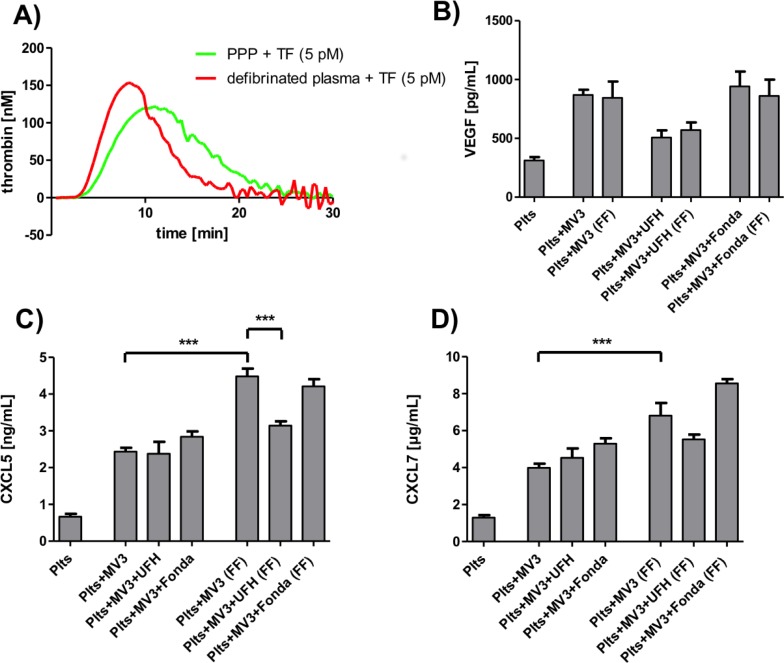
Tumor cell-induced platelet activation in defibrinated plasma. (A) Thrombin generation in recalcified PPP and recalcified, defibrinated PPP due to TF addition. (B) MV3 cell mediated effect on VEGF release in PRP and defibrinated PRP after preincubation with UFH and fondaparinux, respectively, and recalcification (FF, fibrin free plasma). (C) MV3 cell mediated effect on CXCL5 release in PRP and defibrinated PRP after preincubation with UFH and fondaparinux, respectively, and recalcification (FF, fibrin free plasma). (D) MV3 cell mediated effect on CXCL7 release in PRP and defibrinated PRP after preincubation with UFH and fondaparinux, respectively, and recalcification (FF, fibrin free plasma).

To investigate the effect of heparin on platelet activation, which comprises a direct contact mediated and, additionally, a thrombin induced activation, we applied the snake venom batroxobin, to defibrinate plasma. In defibrinated plasma, the coagulation cascade is intact but clot formation will not occur and, thus, concentrations of different mediators can be measured by ELISAs. On that account, both mechanisms for platelet activation–a contact and thrombin dependent–can be investigated simultaneously. As a control, we tested the ability of TF to induce a thrombin formation in defibrinated plasma and found a similar thrombin generation like in PPP (Fig 7A). Then, we activated platelets in defibrinated, recalcified plasma with ADP, TRAP-6 or MV3 cells, respectively, and found identical concentrations of platelet-derived VEGF compared to platelets in PRP where no free CaCl_2_ and thrombin are present (Fig 7B). Thus, the direct contact mediated VEGF release, induced by tumor cells, cannot be further elevated by thrombin. A UFH preincubation of platelets in defibrinated plasma exerted a similar reduction in VEGF secretion compared to UFH treated PRP. Fondaparinux incubation had no impact on VEGF concentration in defibrinated plasma. Next, we quantified the concentration of CXCL5 in defibrinated plasma after MV3 cell administration. Surprisingly, we could detect a significant increase of CXCL5 in defibrinated plasma compared to PRP (Fig 7C). Thus, the CXCL5 release seems to depend on two mechanisms taking place in parallel—a direct contact formation between platelets and MV3 tumor cells and the generation of thrombin. The preincubation of platelets in defibrinated plasma with UFH reduced the CXCL5 concentration to the level of platelets in PRP in which thrombin is not active. Thus, a blockade of the coagulation dependent part of the platelet activation was responsible for the reduced CXCL5 concentrations (Fig 7C). In these experimental settings, fondaparinux had only minor effects on the CXCL5 concentration. Similar to the tumor cell-induced CXCL5 secretion, CXCL7 release was augmented in recalcified, fibrin free plasma in which thrombin formation was feasible (Fig 7D). Here, an UFH preincubation prior to MV3 cell addition led to a mitigated CXCL7 concentration to the level of CXCL7 in PRP. Application of fondaparinux in turn, slightly increased the CXCL7 concentration. These data demonstrate that the tumor cell mediated release of platelets´ chemokines CXCL5 and CXCL7 is regulated by a juxtacrine dependent mechanism and the activation of the coagulation cascade, whereby the coagulation dependent part is susceptible to UFH inhibition (S3 Fig).

## Discussion

In the present study we provide insights into the mechanisms of tumor cell-induced platelet activation in the context of metastatic niche formation and the corresponding therapeutic benefit of heparin. In particular, we addressed two different issues. First, we proved that platelet-derived VEGF release is solely regulated by a direct binding between tumor cells and platelets and can be reduced by heparin application. Therefore, thrombin as the primary product of the plasmatic coagulation cascade seems not to be essential for VEGF secretion. Secondly, discharge of CXCL5 and CXCL7 from platelet granules has been shown to be controlled by direct binding between platelets and tumor cells as well as the plasmatic coagulation cascade. Here, only the coagulation or thrombin dependent part is susceptible to heparin preincubation. This illustrates clearly two modes of action of heparin to interfere with the platelet activation by tumor cells, the attenuation of contacts between cells and the known anticoagulant activity. These results were confirmed by tube formation assays, which simulate the formation of new tumor blood vessels and by granulocyte transmigration assays with platelet releasates. Our results are in line with Battinelli et al. who figured out a shift in platelet mediator release by preincubation of platelets with heparin. Platelets treated with heparin (UFH/low molecular weight heparin (LMWH)) exhibited a reduced VEGF release and an increased release of the anti-angiogenic mediator endostatin after interaction with MCF7 breast cancer cells [27]. The heparin exposure disturbed the angiogenic balance of platelet releasate tending to an antiangiogenic phenotype. Those experiments were conducted in buffer where a VEGF secretion is exclusively mediated by direct binding between tumor cells and platelets. Hence, our data enlarge Battinelli´s findings by incorporation of the coagulation cascade in the experimental settings. In platelets, different mediators are localized in different α-granules and are released separately by different stimuli. Concerning VEGF and endostatin for instance, a discrete storage in α-granules containing either VEGF or endostatin could be exhibited [36,37]. Selective activation of platelet PAR-1 receptor led to an augmented VEGF release whereas a selective PAR-4 receptor activation induced an increased endostatin release accompanied by an unaffected VEGF releasate concentration [36,37]. Recently, the platelet-derived chemokines CXCL5 and CXCL7 were revealed to recruit granulocytes to circulating tumor cells contributing to tumor cell survival, extravasation and finally formation of metastatic foci [26]. The impact of heparin on chemokine release from platelets in the context of metastasis has not yet been investigated. With the application of transmigration experiments using releasates from heparin pretreated platelets we could not detect a reduced migration of purified neutrophils which corroborates our ELISA results and which further shows that different platelet-derived mediators with chemotactic properties (e.g. CCL2, CCL3, CXCL12, or CXCL5) [17] are not significantly reduced by a heparin pretreatment of contact activated platelets. In support of our results, α-granule subpopulations were identified containing different cargos with variable release characteristics depending on specific agonists [36,38]. Using citrated plasma without active thrombin, platelet activation is mediated by interaction with tumor cells and cannot be further enhanced by activation of thrombin PAR-1 receptor by agonists. UFH and LMWH are capable to inhibit the direct binding and adhesion between platelets and tumor cells for instance by inhibition of P-selectin [28,39] but are obviously only partially able to impact the release of different mediators in the early metastatic niche. For platelet-derived chemokine and VEGF release, a short and transient interaction seems to be sufficient in which P-selectin is not involved or dispensable. The induction of mediator release could maybe be mediated by other platelet receptors like CLEC-2, GpVI or GpIb for instance [40]. Utilizing defibrinated plasma, a clear discrimination between contact dependent and thrombin dependent platelet activation and mediator secretion is feasible. Removing of fibrin by batroxobin application revealed no further increase in VEGF release due to tumor cell incubation in recalcified PRP. Hence, the direct interaction between platelets and tumor cells seems to be of key importance for VEGF release, the coagulation cascade in contrary is obviously expendable. In contrast, the chemokine release is affected by both mechanisms and only the thrombin part is reducible by heparin. This is especially true in light of the pronounced TF expression of both tumor cell lines.

TF has long been recognized as key factor for platelet activation in the blood passage of tumor cells [35,41] but for our results, it seems to be relevant especially for chemokine release. For decades, platelets have been regarded as a passive shield for tumor cells in the blood, protecting them from shear stress and the immune system. Meanwhile, it became evident that platelets are of decisive importance for successful metastatic spread of tumor cells. Palumbo and others revealed that platelets downregulate NK cell activity by secreting TGF-β and PDGF (platelet-derived growth factor) or protecting cancer cells from the immune response by camouflaging tumor cells in fibrin-platelet envelopes [19,42,22]. In platelets isolated from tumor-bearing mice, levels of proangiogenic factors were more abundant than in platelets from healthy mice [43,44]. Platelets seem to selectively scavenge and take up angiogenesis regulators in cancer-bearing hosts and these platelets exhibit stronger proangiogenic effects than platelets isolated from cancer-free mice. Furthermore, platelets bound to tumor cells also contribute to cancer cell tethering and arrest under shear flow conditions. These interactions are mediated by P-selectin, integrin α_IIb_β_3_ or tumor cell alpha(v) integrins [45–48].

After firm arrest, platelets have the ability to release metalloproteases (MMPs) from their α-granules at the sites of adhesion leading to increased vascular permeability and elevated tumor cell extravasation [35,49,50]. Growth factors bound to proteoglycans at the vascular endothelium or the ECM are also sequestered. In growing metastatic foci, the continuous supply with oxygen and nutrition is inevitable for a further tumor development. Platelets can stabilize angiogenic tumor blood vessels and efficiently prevent intratumor hemorrhage [51,52]. Finally, platelet-derived TGF-β together with direct binding of platelets to tumor cells can change the tumor cell phenotype from an endothelial to a mesenchymal, a process which can confer stem cell like properties to cancer cells [23]. The impact of heparin or other anticoagulants on the release characteristics of these mediators warrants further investigations. Nonetheless, with the present study, we reveal how heparin affects the release of VEGF and chemokines CXCL5 and CXCL7 in the early metastatic spread of tumor cells.

## Conclusions

The efficient interference in the tumor cell platelet communication could comprise great therapeutic opportunities since a lot of decisive steps in the metastatic cascade are expedited by platelets. In this context, heparin, which is routinely administered to cancer patients, has a certain impact because some of the protumorigenic effects are inhibited or at least decelerated. In several clinical trials heparin has revealed to prolong cancer patients overall survival whereas in other studies no benefit for patients was detectable [53–55]. Currently, some clinical trials, examining the effect of heparin for tumor patients, are ongoing. Since several years, novel oral anticoagulants as an alternative for heparin are applied to patients suffering from venous thromboembolism. The effect of these new classes of drugs on metastasis in cancer patients is uncertain since the molecular structure differs from heparin´s structure. Thus, positive side effects, which are not related to thrombin or factor Xa inhibition are questionable for these drugs. In the future, in-depth studies, enquiring the exact mechanisms by which platelets release different mediators due to different activators or contacts to different cells, are eligible. Also a comparison of a LMWH with the novel oral anticoagulants concerning the impact on the platelet secretome induced by tumor cells could be an interesting approach since LMWH exhibits a heparin structure and inhibits thrombin and factor Xa. Corresponding results may further elucidate the impact of heparin on mediator release in the formation of the early metastatic niche.

## Supporting information

S1 Fig (A) VEGF release induced by U937 monocytic cells was quantified by ELISA. (B) Transmigration of granulocytes was determined in transwell chambers for 16 h. In the lower part of the chamber was plasma from platelets activated with MV3 or MCF7 cells and incubated with UFH as indicated. Freshly isolated granulocytes in RPMI medium were placed in the upper part of the chamber. Luminescence was quantified in a plate reader. (C) Thrombin generation in recalcified PPP of MV3 and MCF7 cell supernatant was determined and compared to TF (1 pM and 5 pM). (D) TF expression on MV3 and MCF7 cells was determined by flow cytometry using a FITC-labeled anti human TF mAb.
(EPS)

S2 Fig Impact of P-selectin inhibition on platelet tumor cell interaction and VEGF release. (A) Adhesion of MV3 melanoma cells to Calcein-AM labeled platelets activated with ADP or TRAP-6, respectively, was quantified with a plate reader. Where indicated anti-human P-selectin mAb was added to the platelets. (B) Platelets in citrated plasma, preincubated with anti-human P-selectin mAb, were incubated either with MV3 melanoma or MCF7 breast cancer cells and VEGF release was quantified by ELISA.
(EPS)

S3 Fig Schematic overview of the heparin mediated effects on the platelet tumor cell communication. Contact dependent VEGF release from platelets is reduced by heparin application whereas the contact induced chemokine release is not affected. The chemokine release (CXCL5 and CXCL7) is elevated when thrombin is present and can be reduced by heparin.
(EPS)
